# Clinicopathological features and lymph node and distant metastasis patterns in patients with gastroenteropancreatic mixed neuroendocrine‐non‐neuroendocrine neoplasm

**DOI:** 10.1002/cam4.4031

**Published:** 2021-06-10

**Authors:** Panpan Zhang, Zhongwu Li, Jie Li, Jian Li, Xiaotian Zhang, Zhihao Lu, Yu Sun, Yan Li, Jun Zhou, Xicheng Wang, Zhi Peng, Lin Shen, Ming Lu

**Affiliations:** ^1^ Department of Early Drug Development Centre Key Laboratory of Carcinogenesis and Translational Research Ministry of Education Peking University Cancer Hospital and Institute Beijing China; ^2^ Department of Pathology Key Laboratory of Carcinogenesis and Translational Research Ministry of Education Peking University Cancer Hospital and Institute Beijing China; ^3^ Department of Gastrointestinal Oncology Key Laboratory of Carcinogenesis and Translational Research Ministry of Education Peking University Cancer Hospital and Institute Beijing China

**Keywords:** distant metastasis, gastroenteropancreatic, lymph nodes metastasis, mixed adenoneuroendocrine carcinoma, mixed neuroendocrine‐non‐neuroendocrine neoplasm

## Abstract

**Objective:**

Owing to its rarity and heterogeneity, the biological behavior and optimal therapeutic management of mixed neuroendocrine‐non‐neuroendocrine neoplasm (MiNEN) have not been established. Herein, we aimed to evaluate the clinicopathological characteristics and metastatic patterns of MiNEN.

**Methods:**

Continuous clinicopathological data of MiNEN patients treated at our hospital were retrospectively collected and analyzed.

**Results:**

This study had enrolled 169 patients since January 2010 to January 2020. Pathological components were assessed in 129 patients with MiNEN (76.3%), and a focal (non‐)neuroendocrine component was observed in 40 patients (23.7%; <30% of the tumor). Among the enrolled patients, 80 underwent surgical removal of the primary tumor and lymph nodes (LNs), and 34 with distant metastasis underwent biopsy of both primary tumor and metastatic lesions. In patients with LN metastasis, 68.8% (55/80) exhibited a pure component of either neuroendocrine (NE) or adenocarcinoma/squamous carcinoma (AS) in metastatic LNs, while 20% (16/80) showed different components in different LNs, and only 11.2% (9/80) exhibited both NE and AS components in the same LN. In patients with distant metastases, 26.5% (9/34) possessed coexisting NE and AS components in the distant metastases, 70.6% (24/34) were regarded as a pure NE component, and 2.9% (1/34) were comprised of a pure AS component.

**Conclusion:**

Lymph node and distant metastases exhibited distinct metastatic patterns in patients with MiNEN. The major pathological component in regional LNs may have influenced the proportion of the two components within the primary tumor, but distant metastases were dominated by the NE component.

## Graphical abstract

Regional lymph nodes and distant metastases of MiNEN exhibited distinct metastatic patterns. We recommend that patients with MiNEN undergo aggressive multidisciplinary oncologic management, and an optimal modality should be established based on the NEC component.

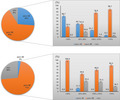

## 1 INTRODUCTION

Gastroenteropancreatic tumors involving both exocrine and neuroendocrine components are exceedingly rare and were first reported by Cordier in 1924.^1^ These two components can exhibit various morphological patterns and form mixed neoplasms, which are classified into three categories: collision, composite, and amphicrine tumors.^2^ One subtype of neuroendocrine carcinoma with both epithelial and neuroendocrine cells, the mixed adenoneuroendocrine carcinoma (MANEC) was defined by the World Health Organization (WHO) at 2010, with the epithelial and neuroendocrine component each comprising at least 30% of the tumor.^3^ The term MANEC, however, does not adequately convey the morphological heterogeneity of the disease and has caused confusion in the literature. In the latest version of WHO classification in 2017, MANEC was renamed as mixed neuroendocrine‐non‐neuroendocrine neoplasm (MiNEN).^4^ Owing to its rarity, morphologic diversity, and distinct primary origins, the origin and classification of this neoplasm are not well‐defined, and the optimal therapeutic management has not yet been established. It remains unclear whether MiNEN should be treated as an adenocarcinoma or a neuroendocrine neoplasm.

To gain a better understanding of the heterogeneous nature of MiNEN and to preliminarily establish guidelines for reasonable clinical management, we retrospectively investigated the clinicopathological features and metastatic patterns of MiNEN treated at our center. To our knowledge, this is one of the largest single‐center MiNEN cohorts to date, as well as the first extended report concerning metastatic characteristics of this rare tumor type. Findings from this study can contribute to better understanding the biological behavior of this disease, thereby facilitate better treatment decisions.

## 2 PATIENTS AND METHODS

### 2.1 Patients

We retrospectively collected clinical, pathological and survival data of patients diagnosed with gastroenteropancreatic neuroendocrine neoplasm (GEP‐NEN) at our hospital from January 2010 to January 2020. The pathology database was further explored to identify patients with a pathological diagnosis of MANEC or MiNEN, defined as each tumor component accounting for at least 30% of the tumor, based on the 2010 and 2017 WHO classifications. Patients with two pathological components who failed to meet the criteria (each component accounting for less than 30% of the tumor) were also included in the study. Tumor stages were assigned according to the AJCC 8^th^ Edition Cancer Staging system.

**FIGURE 1:**
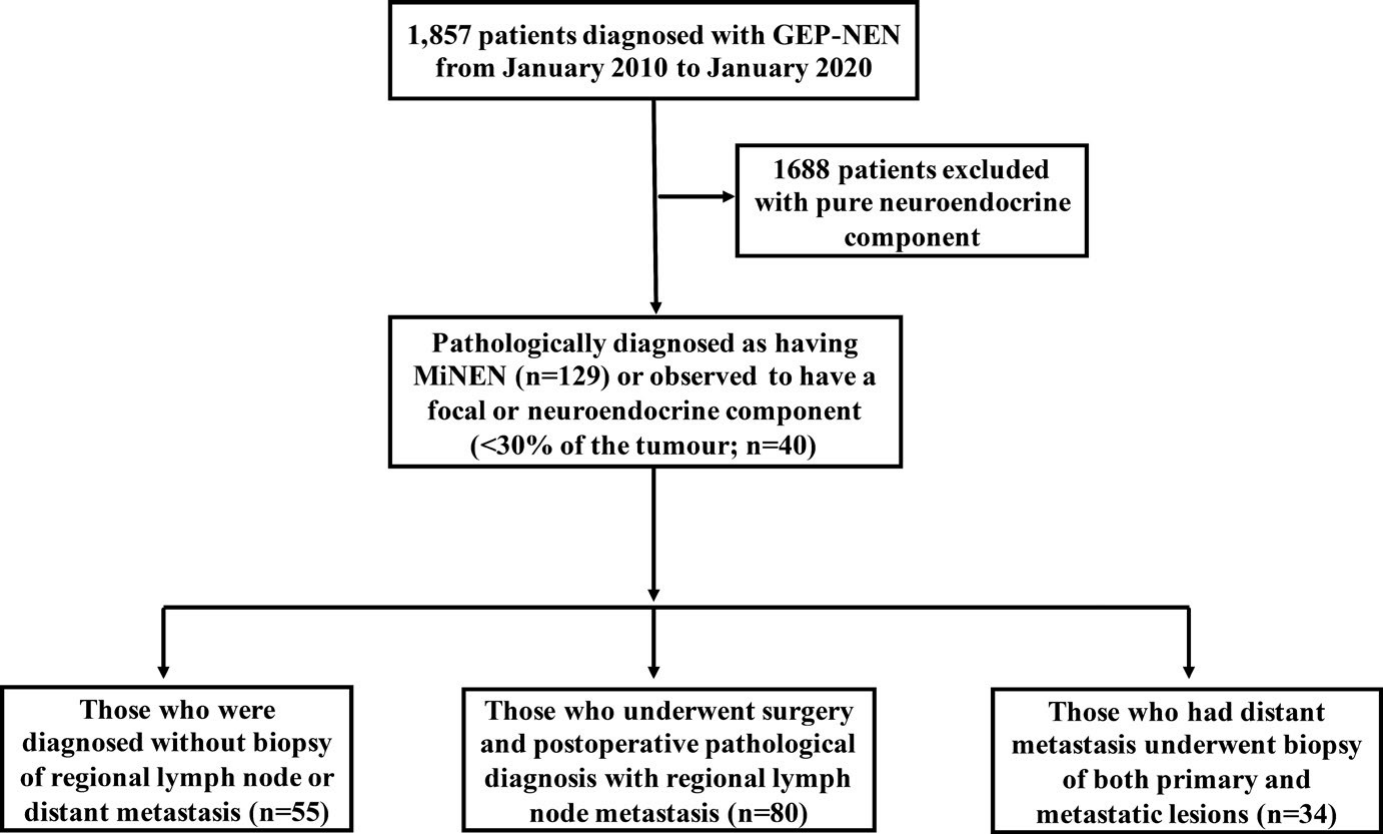
Flow diagram of study selection. n = number of patients; MiNEN = mixed neuroendocrine non‐neuroendocrine neoplasm

For the analysis of pathological component patterns in lymph nodes, we enrolled patients who underwent surgery for primary tumor and lymph node dissection. For the analysis of distant metastases, those who had distant metastasis at diagnosis or recurrence and underwent biopsy of both primary and metastatic lesions were enrolled. The flow diagram of the study is shown in Figure 1. This study was approved by our institutional research ethics board. All enrolled patients provided written consent for usage of their information and specimens stored in the hospital database for research.

### 2.2 Diagnosis and classification

According to the 2017 WHO classification, MiNEN is defined as a malignant tumor containing at least 30% of both glandular epithelial cells and neuroendocrine cells. Patients with tumors that had two components but did not meet the criteria of MiNEN were also enrolled, and their tumors were assigned to either a neuroendocrine (NE)‐dominant type or an adenocarcinoma/squamous carcinoma (AS)‐dominant type according to the tumor dominance. For each case, all slides of the primary tumor, regional lymph nodes and/or distant metastases were reviewed, and the following parameters were collected: the proportion and histologic subtypes of the two components, the number of mitoses per 10 high‐power fields (HPFs), the Ki67 index of the neuroendocrine component, and the number and content of metastasis. All pathological findings were reviewed and confirmed by two pathologists independently.

Based on the histological type, regional lymph nodes and/or distant metastases, each tissue sample was assigned into one of the following groups: (1) tissues with a neuroendocrine carcinoma or adenocarcinoma (or squamous carcinoma) component only; (2) tissues exhibiting coexistence of adenocarcinoma (or squamous carcinoma) and neuroendocrine carcinoma within one metastatic lesion; and (3) tissues with distinct metastatic lesions showing either an adenocarcinoma (or squamous carcinoma) or a neuroendocrine carcinoma component exclusively.

### 2.3 Statistical analysis

The Student's *t* test, χ^2^ test (or Fisher's exact test) and the Mann‐Whitney test were used to analyze the clinicopathological characteristics of the patients. Overall survival (OS) time was measured from the date of initial diagnosis until the date of death or the date of the last known follow‐up. The log‐rank test was used to compare survival rates. Statistical tests used two‐tailed P values, and *p* < 0.05 was considered statistically significant. All statistical analyses were performed with SPSS (version 25; IBM).

## 3 RESULTS

### 3.1 Clinicopathological characteristics

**FIGURE 2:**
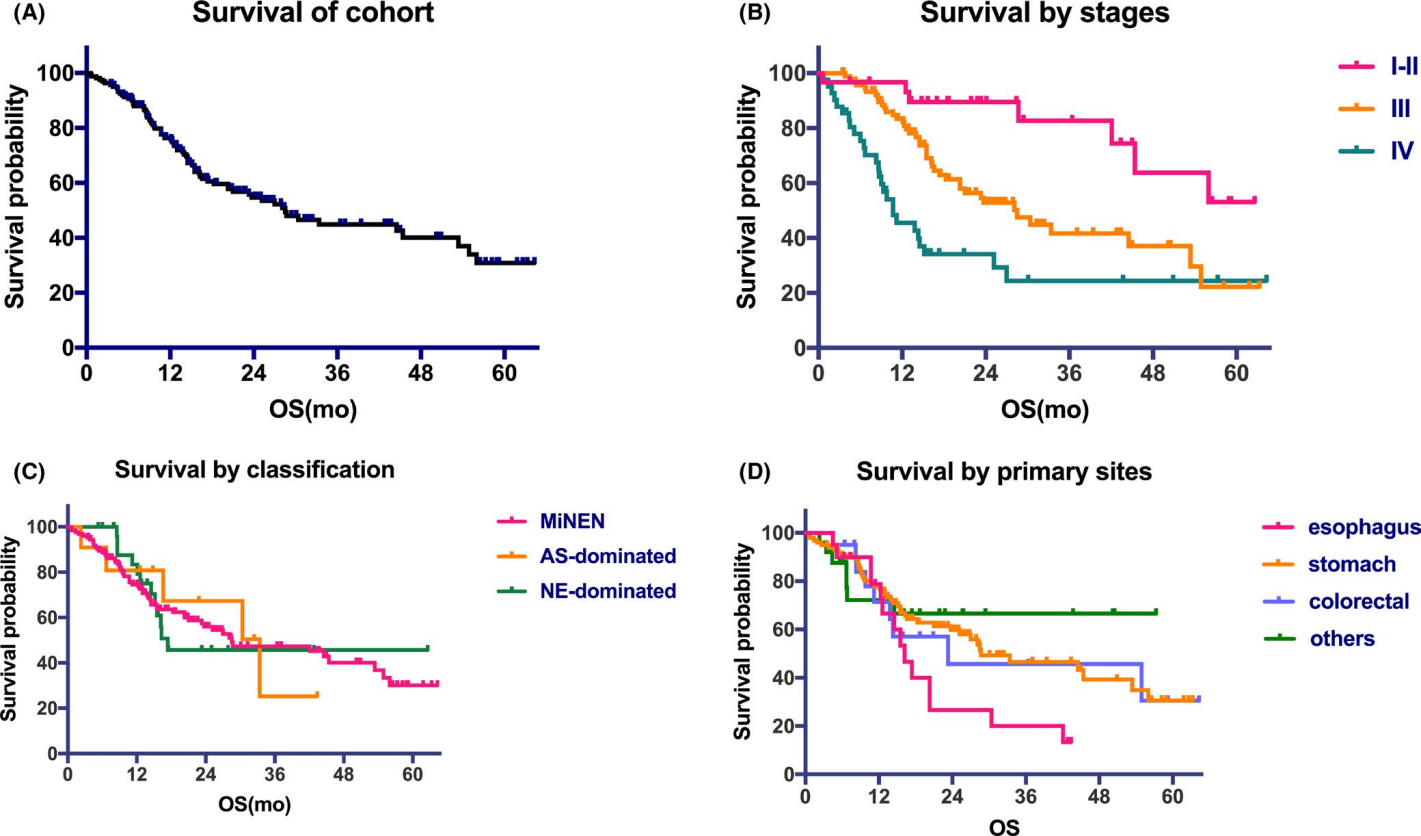
Overall survival curve of the cohort (A) and survival curves based on stages (B), classification (C) and primary sites (D)

The clinicopathological data of patients treated at our hospital from January 2010 to January 2020 were reviewed, and data of 1,857 patients with a pathologic diagnosis of GEP‐NEN were retrieved. Among these patients, 169 with both glandular epithelial and neuroendocrine cells were identified, and 129 of these patients (76.3%) were classified as patients with MiNEN, in line with the 2017 WHO classification. In the remaining 40 (23.7%) cases, a focal (non‐)neuroendocrine component (<30% of the tumor tissue) was observed. Among the enrolled patients, 27/169 (16%) were diagnosed by biopsy only, and the rest underwent operative pathological diagnosis. The primary tumors were identified in the following sites: the stomach (104, 61.5%), colorectal (21, 12.4%), esophagus (19, 11.2%), duodenum (7, 4.1%), biliary tract (6, 3.6%), pancreas (5, 3%), and other sites (7, 4.1%). The clinicopathological characteristics of the entire cohort (n = 169) are shown in Table 1. For AS component, there were 22 cases (13.0%) of squamous carcinoma and 147 (87.0%) adenocarcinomas of varying degrees of differentiation. For the NE component, there were 6 patients (3.6%) with neuroendocrine tumor (NET) and 163 patients (96.4%) with neuroendocrine carcinoma (NEC). Patients in stage I‐II, III and IV exhibited 3‐year survival rates of 82%, 41% and 25%, respectively; and their median OS (mOS) were not available (NA), 28.5 and 10.7 months (*p* < 0.0001), respectively. There was no difference in survival outcomes among patients with MiNEN, AS‐dominant and NE‐dominant tumors (*p* = 0.91). The mOS of patients with primary esophageal tumors (16.2 months) or colorectal primary tumors (23.3 months) was shorter than that of patients with primary stomach tumors (28.7 months) and primary tumors at other primary sites (NA), but the difference was not statistically significant (*p* = 0.14) (Figure 2).

**TABLE 1:** Clinicopathological characteristics of patients with a diagnosis of MiNEN and those with NE‐dominated and AS‐dominated tumors

Characteristics, n (%)	MiNEN (n = 129)	NE‐dominant (n = 29)	AS‐dominant (n = 11)	All (n = 169)
Age (year)				
<60	57 (44.2)	14 (48.3)	1 (9.1)	72 (42.6)
≥60	72 (55.8)	15 (51.7)	10 (90.9)	97 (57.4)
Gender				
Male	108 (83.7)	27 (93.1)	9 (81.8%)	144 (85.2)
Female	21 (16.3)	2 (6.9)	2 (18.2%)	25 (14.8)
Tumor location				
Esophagus	13 (10.1)	5 (17.2)	1 (9.1)	19 (11.2)
Stomach	78 (60.5)	19 (65.5)	7 (63.6)	104 (61.5)
Duodenum	5 (3.9)	0 (0.0)	2 (18.2)	7 (4.1)
Pancreas	4 (3.1)	1 (3.4)	0 (0.0)	5 (3.0)
Colorectum	19 (14.7)	2 (6.9)	0 (0.0)	21 (12.4)
Biliary tract	4 (3.1)	1 (3.4)	1 (9.1)	6 (3.6)
Others	6 (4.7)	1 (3.4)	0 (0.0)	7 (4.1)
TNM stage				
I‐II	22 (17.1)	4 (13.8)	4 (36.4)	30 (17.8)
III	68 (52.7)	22 (75.9)	5 (45.4)	95 (56.2)
IV	39 (30.2)	3 (10.3)	2 (18.2)	44 (26.0)

AS, adenocarcinoma/squamous carcinoma; n, number of patients; NE, neuroendocrine.

Detailed pathological evaluation of the components of lymph node metastasis was available in 80 patients who underwent the primary tumor and regional lymph node resection. In this cohort, 60 patients were classified as MiNEN, 18 were NE‐dominant type (>70% of tumor tissue), and 2 were AS‐dominant type. There were 34 patients who had distant metastases at diagnosis or recurrence and biopsies of the metastatic lesion were available for evaluation. This cohort contained 25 patients with MiNEN, 6 patients with NE‐dominant type tumors and 3 patients with AS‐dominant type tumors.

### 3.2 Lymph node metastatic patterns

This cohort included 80 patients, and 55 patients (68.8%) exhibited lymph nodes with a pure component, 16 patients (20%) had different components in different LNs, and only 9 patients (11.2%) had a mixture of two components coexisting in the same lymph node. In cases with a pure component in the lymph nodes (n = 55), 13 had a pure AS component and 42 had a pure NE component. The metastatic patterns of the lymph nodes are shown in Table 2.

**TABLE 2:** Correlation of the proportion of NE component or AS differentiation in primary tumor sites with regional lymph node metastatic patterns in 80 patients

Characteristics, n (%)	Pure NE	Pure AS	Mixed component	
			Different LN^a^	Coexisting in same LN^b^
NE proportion				
<30%	0 (0)	1 (50.0)	1 (50.0)	0 (0)
30%−50%	11 (35.5)	8 (25.8)	7 (22.6)	5 (16.1)
>50% ≤70%	18 (62.1)	3 (10.3)	6 (20.7)	2 (6.9)
>70%	13 (72.2)	1 (5.6)	2 (11.1)	2 (11.1)
AS differentiation				
Poorly differentiated	19 (50)	4 (10.5)	9 (23.7)	6 (15.8)
Well or moderated	23 (54.8)	9 (21.4)	7 (16.7)	3 (7.1)
Total	42 (52.5)	13 (16.3)	16 (20)	9 (11.2)

AS, adenocarcinoma/squamous carcinoma; LN, lymph node; n, number of patients; NE, neuroendocrine.

^a^ Two components separately metastasizing to different lymph nodes.

^b^ Mixture of two components coexisting in the same lymph nodes.

**FIGURE 3:**
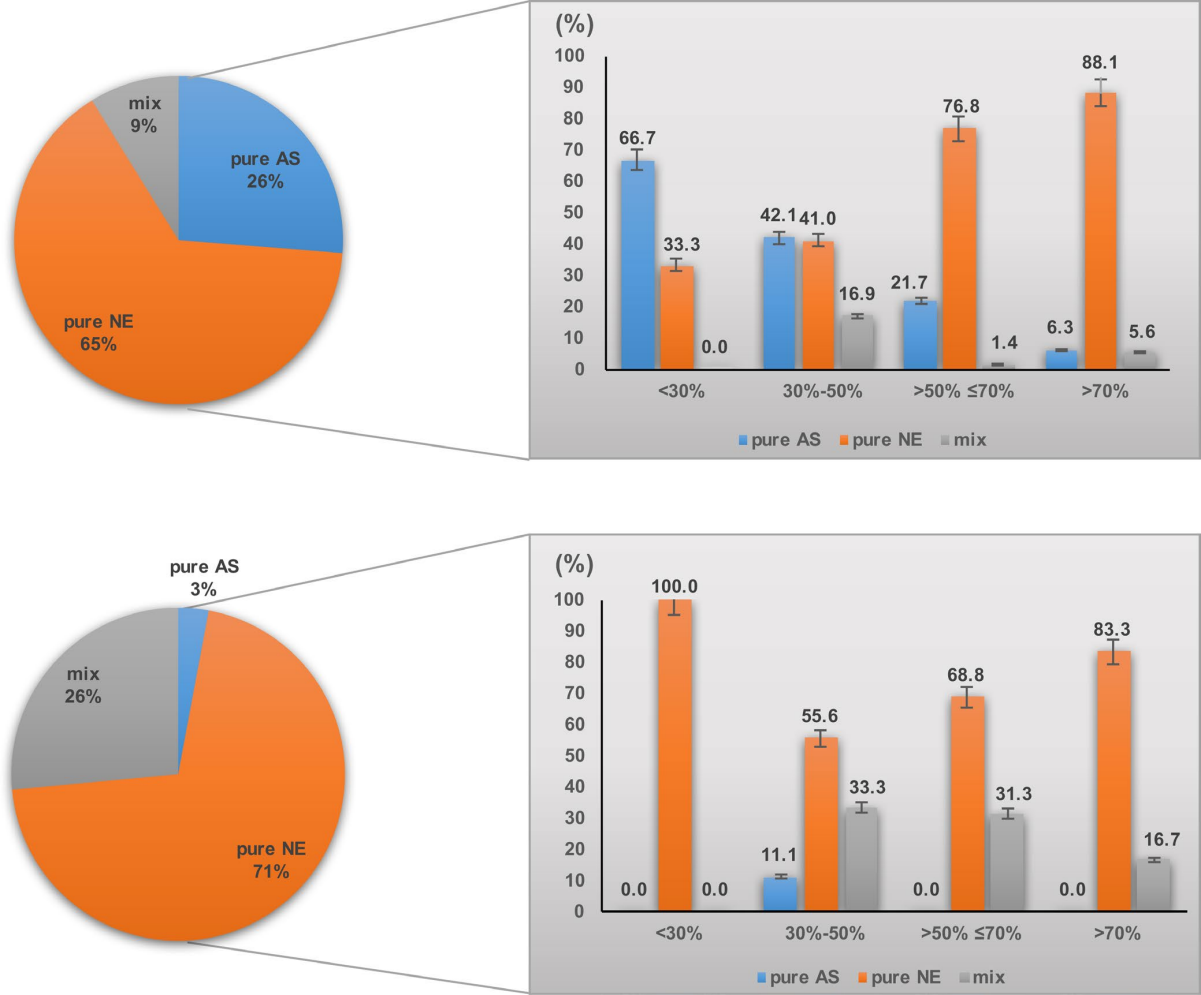
Subgroup analysis classified by the percentage of NE component in the primary site. The upper panels show the components of 453 lymph node metastasis, and the lower panels show the components of 34 distant metastasis. NE, neuroendocrine; AS, adenocarcinoma/squamous carcinoma; LN, lymph node

In these 80 patients, a total of 453 lymph node metastases were identified, including 119 (26.3%) with a single AS component, 294 (64.9%) with pure NE component and 40 (8.8%) with a mixture of components containing NE and AS. Lymph node metastatic patterns may be associated with the proportion of two components within the primary tumors (Figure 3). Subgroups were classified by the percentage of NE component (<30%, 30%‐50%, >50% ≤70% and >70%) within the primary tumor sites. The ratio of lymph nodes with a single AS component decreased to 66.7%, 42.1%, 21.7% and 6.3%; while the proportion with a pure NE component increased to 33.3%, 41.0%, 76.8% and 88.1%, respectively.

### 3.3 Distant metastatic patterns

Among the 34 patients with distant metastases at diagnosis or recurrence, 24 metastatic tumors (70.6%) consisted of a single NE component (1 NET G2 and 23 poorly differentiated NEC), 9 patients (26.5%) showed coexistence of both NE and AS components, and only 1 patient (2.9%) had a pure adenocarcinoma component. There were 24 patients with liver metastases in this cohort, 19 of which comprised of a pure NE component, 4 were made up by a mixture of both AS and NE components, and only 1 with a pure adenocarcinoma component. Among the 8 patients with peritoneal or supraclavicular lymph node metastases, 4 possessed a pure NE component, and the remaining lymph node metastases demonstrated the properties of a mixture of both AS and NE components. The remaining 2 metastases were in the lung (n = 1), containing both AS and NE components, and in the brain (n = 1), with a pure NE component, respectively.

As for the pathological degree of differentiation, in patients with a primary tumor with a poorly differentiated AS component (n = 13), the metastatic lesions were invaded by a pure NE component in 10 cases, by a pure AS component in 1 case, and by mixed components in 2 cases. Among patients with well or moderately differentiated AS components (n = 21), 14 had a pure NE component within distant metastasis, while the remaining 7 patients had distant lesions revealing coexistence of both NE and AS components.

We also enrolled 9 patients with a focal (non‐)neuroendocrine component less than 30% of the tumor; these patients presented a domination by NE (n = 6) or AS (n = 3) in the primary tumors. In 8 of these patients (88.9%), distant metastases showed a pure NE component, while the coexistence of both AS and NE components were observed in 1 patient. Of note, in all 3 cases with a NE component accounting for less than 30% of the primary tumor, metastases were invaded by a pure NE component (Table 3).

**TABLE 3:** Correlation of the percentage of NE component in primary tumor sites with a distant metastatic pattern in 34 patients

Characteristics, n (%)	Pure NE	Pure AS	Mixed component
Synchronic	13 (65)	1 (5)	6 (30)
Non‐synchronic	11 (78.6)	0 (0)	3 (21.4)
NE proportion			
<30%	3 (100)	0 (0)	0 (0)
30%−50%	5 (55.6)	1 (11.1)	3 (33.3)
>50% ≤70%	11 (68.8)	0 (0)	5 (31.2)
>70%	5 (83.3)	0 (0)	1 (16.7)
Metastatic sites			
Liver	19 (79.2)	1 (4.3)	4 (17.4)
Lung or brain	1 (50)	0 (0)	1 (50)
Distant lymph nodes^a^	4 (50)	0 (0)	4 (50)
Total	24 (70.6)	1 (2.9)	9 (26.5)

AS, adenocarcinoma/squamous carcinoma; LN, lymph node; n, number of patients; NE, neuroendocrine.

^a^ Distant lymph nodes indicate supraclavicular or peritoneal nodules.

## 4 DISCUSSION

Due to the rarity and heterogeneity of MiNEN, its location‐specific clinical features, pathological classification, and metastatic patterns are not well understood. In the present study, the most common primary site of MiNEN was the stomach and colorectum. When defining MiNEN, the 2017 and 2019 versions of the WHO classification criteria used more general terms to include histological variants of squamous carcinoma, pancreatic ductal adenocarcinoma or low‐grade NET as one or both components. In this study, the majority of exocrine components observed were adenocarcinoma, and the majority of NE components were NEC. Therefore, MiNEN is an aggressive entity with poorly differentiated NEC components in most cases and is thus associated with poor prognosis. Furthermore, only 16% of the patients (27/169) were diagnosed by biopsy only; the diagnosis of the rest of the patients was confirmed through postoperative pathology. Thus, it is difficult to diagnose MiNEN only with biopsy, and special attention should be paid to the potential bias in evaluating biopsy specimens. Therefore, it is recommended that pathological specimens should be evaluated after the surgical removal of the entire neoplasm.

Current WHO classification criteria requires that each component of MiNEN accounts for at least 30% of the entire tumor, which was originally proposed by Lewin in 1987.^2^ It was speculated that the prognosis of MiNEN was affected by major histological factors rather than the components accounting for <30% of the entire tumor. Currently, the threshold in this definition becomes controversial and has caused confusion in therapeutic management of these neoplasms. In the literature, Jiang et al^5^ set the volume threshold as ≥20% for the NE component. In addition, Park et al^6^ indicated that survival of patients with a NE component of greater than 10% was poorer than that of their counterparts, but no survival difference was observed between NEC and MiNEN patients. In our study, no significant survival difference was observed among MiNEN, NE‐dominated and AS‐dominated patients. However, we found that even if the NE component comprised less than 30% of the primary tumor, a pure NE component could still appear in distant metastases. Due to the rarity of such mixed neoplasms, our findings are valuable and highlight the need for optimal thresholds and criteria for assessing the biological behavior and treatment response. In clinical management, the percentage of each component must be clarified; and for biopsy specimens with NE component <30%, the heterogeneity of the neoplasm needs to be considered to develop a comprehensive treatment plan.

MiNEN is frequently diagnosed with extensive lymph node and liver metastasis, which is the most important risk factor for poor prognosis. However, there is currently no consensus on the metastatic patterns in the literature. In the current study, MiNEN cases were grouped according to the ratio of primary tumor components, and the metastatic lymph nodes and distant lesions of each patient were evaluated for their pathological components. As the proportion of the NE component in the primary tumor increased, the ratio of positive lymph nodes with pure NE invasion in each group also increased. Previous case reports have revealed that lymph nodes and liver metastases are usually invaded by a neuroendocrine carcinoma component rather than an adenocarcinoma component.^7^, ^8^, ^9^, ^10^ Watanabe et al.^11^ reported that pathological analysis of metastasized tissue in 9 patients with recurrent MiNEN demonstrated an NE component in 5 patients and an AC component in 4 patients. In our study, the results revealed that both regional lymph nodes and distant metastases were primarily invaded by one component. However, distant metastases were primarily invaded by the NE component. Moreover, 26.5% of patients had a mixture of both AS and NE components; and the pure exocrine component rarely metastasized to distant organs. Therefore, the proportion of components within the primary lesion provide limited information regarding adjuvant treatment regimen choices for MiNEN patients. Meanwhile, the presence of an NE component in the original neoplasms increases the chance of recurrence in distant metastases.

In clinical practice, the biological behavior and prognostic factors of MiNEN are controversial. At present, there are two main opinions in the field: (1) It has been proposed that the volume proportion of the two components determines the clinical course of MiNEN. Chen et al^12^ regarded high volume (>50%) of a high‐grade NE component as an independent poor prognostic factor in patients with MiNEN. It was hypothesized that the prognosis is influenced by the predominant histological component rather than the one accounting for <30% of the entire neoplasm.^13^ (2) Other researchers have proposed that treatment should target the more aggressive component within the tumor, regardless of proportion. They argue that a minor poorly differentiated NEC component can impact prognosis, as recent studies have demonstrated that the prognosis of MiNEN is defined by the more invasive component.^14^, ^15^ As previous research data have been extremely heterogeneous in terms of primary tumor site, disease stage and type of information provided, our study is the largest retrospective analysis of GEP MiNEN examining lymph node and distant metastatic patterns. Our results revealed that there is a clear correlation between regional lymph node metastasis component and the proportion of each component within the primary tumor. Of note, the NE component tended to metastasize, and tumors with an NE component comprising less than 30% volume of the mixed tumors could still occur in distant metastasis and exert an impact on the prognosis.

The cause of different metastatic patterns of lymph nodes and distant metastases, as well as the intrinsic forces that drives the two components to metastasize, remain unclear. An increasing number of pathologists have made efforts to identify the histological origin of the mixed carcinoma. It is proposed that MiNEN originates from single endoderm pluripotent stem cells, which are affected by hormones, the microenvironment and unstable genomes during the process of tumor development, resulting in two‐way or multidirectional differentiation.^16^ However, another theory proposed that the two components of MiNEN descend from two different cell linages.^17^ However, the oncogenesis and malignant behavior in this tumor type are far from settled. Molecular studies have reported that NE components share molecular abnormalities with their adenocarcinoma counterparts while also displaying additional alterations.^18^, ^19^, ^20^ In the present study, we found that distant lesions were usually invaded by pure NE or mixed components. By comparison of synchronic and non‐synchronic metastases, we found that the proportion of coexisting components increased in lesions that relapsed during the course of disease. Further studies should focus on molecular mechanisms to reveal the intrinsic driving forces of metastatic patterns and to identify the pathogenic pathways involved in the carcinogenic progress.

To date, no clinical practice guidelines have been developed, and only a limited number of case series on MiNEN have been published. Surgical resection is considered as the only curative treatment, and adjuvant chemotherapy is recommended due to the high recurrence rate of these tumors.^21^ MiNEN is often diagnosed at an advanced stage as a result of its aggressive nature and may not be suitable for curative resection. In such cases, chemotherapy with cytotoxic drugs plays a primary role in the treatment. However, it is controversial to establish universal guidelines for adjuvant and palliative chemotherapy in MiNEN patients. In our study, we found that distant metastatic lesions that occurred at diagnosis or recurrence had either pure NEC or coexisting component(s). Therefore, the NE component of MiNEN is most likely the primary cause of the malignancy of the disease, regardless of the proportion of the two components in the primary tumor. We recommend that patients with MiNEN undergo aggressive multidisciplinary oncologic management, and an optimal modality should be established based on the NEC component.

## 5 CONCLUSION

Collectively, MiNEN is a heterogeneous disease that has been overlooked. Regional lymph nodes metastasis might affect the proportion of different components in the primary tumor, while distant metastasis is primarily caused by NE or coexisting components. The NE component primarily affects invasion or recurrence in distant metastases and should be considered when deciding the appropriate treatment for MiNEN. Moreover, an NE component of <30% volume of the mixed primary tumor can still metastasize and affect prognosis, indicating that the diagnosis criteria for MiNEN should be further investigated.

## CONFLICT OF INTEREST

The authors declared no potential conflicts of interest with respect to the research, authorship, and publication of this article.

## AUTHOR CONTRIBUTIONS

Panpan Zhang, Zhongwu Li and Ming Lu designed the research. Panpan Zhang wrote the manuscript and analyzed the data. Zhongwu Li, Jie Li, Jian Li, Xiaotian Zhang, Zhihao Lu, Yu Sun, Yan Li, Jun Zhou, Xicheng Wang, Zhi Peng and Lin Shen collected the data and technical information. Ming Lu reviewed and helped revise the manuscript. All authors approved the final proof.

## Data Availability

The datasets used and/or analyzed during the current study are available from the corresponding author on reasonable request.

## References

[1] Cordier R . Les cellules argentaffines dans les tumeurs intestinales. Arch Int Med Exp. 1924;1(5).

[2] Lewin K . Carcinoid tumors and the mixed (composite) glandular‐endocrine cell carcinomas. Am J Surg Pathol. 1987;11(Suppl 1):71‐86. 3544888 10.1097/00000478-198700111-00007

[3] Bosman F . World Health Organization International Agency for Research on Cancer. WHO classification of tumours of the digestive system. 2010:160‐166.

[4] de Mestier L , Cros J , Neuzillet C , et al. Digestive system mixed neuroendocrine‐non‐neuroendocrine neoplasms. Neuroendocrinology. 2017;105(4):412‐425. 10.1159/000475527. 28803232

[5] Jiang S‐X , Mikami T , Umezawa A , Saegusa M , Kameya T , Okayasu I . Gastric large cell neuroendocrine carcinomas: a distinct clinicopathologic entity. Am J Surg Pathol. 2006;30(8):945‐953. 16861964 10.1097/00000478-200608000-00003

[6] Park JY , Ryu M‐H , Park YS , et al. Prognostic significance of neuroendocrine components in gastric carcinomas. Eur J Cancer. 2014;50(16):2802‐2809. 25201164 10.1016/j.ejca.2014.08.004

[7] Zhang W , Xiao W , Ma H , Sun M , Chen H , Zheng S . Neuroendocrine liver metastasis in gastric mixed adenoneuroendocrine carcinoma with trilineage cell differentiation: a case report. Int J Clin Exp Pathol. 2014;7(9):6333. 25337287 PMC4203258

[8] Xenaki S , Lasithiotakis K , Andreou A , et al. A rare case of mixed neuroendocrine tumor and adenocarcinoma of the pancreas. Case Rep Surg. 2016;2016:1‐4. 10.1155/2016/3240569 PMC5005596 27610261

[9] Max N , Rothe A , Langner C . Mixed adenoneuroendocrine carcinoma of the ampulla of Vater: a case report. Mol Clin Oncol. 2016;5(1):95‐98. 27330774 10.3892/mco.2016.858 PMC4906782

[10] Zhang L , DeMay RM . Cytological features of mixed adenoneuroendocrine carcinoma of the ampulla: two case reports with review of literature. Diagn Cytopathol. 2014;42(12):1075‐1084. 24554593 10.1002/dc.23107

[11] Watanabe J , Suwa Y , Ota M , et al. Clinicopathological and prognostic evaluations of mixed adenoneuroendocrine carcinoma of the colon and rectum: a case‐matched study. Dis Colon Rectum. 2016;59(12):1160‐1167. 27824701 10.1097/DCR.0000000000000702

[12] Chen M‐H , Kuo Y‐J , Yeh Y‐C , et al. High neuroendocrine component is a factor for poor prognosis in gastrointestinal high‐grade malignant mixed adenoneuroendocrine neoplasms. J Chin Med Assoc. 2015;78(8):454‐459. 26002564 10.1016/j.jcma.2015.04.002

[13] Brathwaite S , Rock J , Yearsley MM , et al. Mixed adeno‐neuroendocrine carcinoma: an aggressive clinical entity. Ann Surg Oncol. 2016;23(7):2281‐2286. 26965701 10.1245/s10434-016-5179-2 PMC4891253

[14] Lee EJ , Park SM , Maeng L , Lee A , Kim KM . Composite glandular‐endocrine cell carcinomas of the stomach: clinicopathologic and methylation study. Apmis. 2005;113(9):569‐576. 16218931 10.1111/j.1600-0463.2005.apm_190.x

[15] Lee HH , Jung CK , Jung ES , Song KY , Jeon HM , Park CH . Mixed exocrine and endocrine carcinoma in the stomach: a case report. J Gastric Cancer. 2011;11(2):122‐125. 22076213 10.5230/jgc.2011.11.2.122 PMC3204483

[16] Jain D , Eslami‐Varzaneh F , Takano AM , et al. Composite glandular and endocrine tumors of the stomach with pancreatic acinar differentiation. Am J Surg Pathol. 2005;29(11):1524‐1529. 16224221 10.1097/01.pas.0000169498.89035.f9

[17] Mondolfi AEP , Slova D , Fan W , et al. Mixed adenoneuroendocrine carcinoma (MANEC) of the gallbladder: a possible stem cell tumor? Pathol Int. 2011;61(10):608‐614. 21951672 10.1111/j.1440-1827.2011.02709.x

[18] Scardoni M , Vittoria E , Volante M , et al. Mixed adenoneuroendocrine carcinomas of the gastrointestinal tract: targeted next‐generation sequencing suggests a monoclonal origin of the two components. Neuroendocrinology. 2014;100(4):310‐316. 10.1159/000369071. 25342539

[19] Vanacker L , Smeets D , Hoorens A , et al. Mixed adenoneuroendocrine carcinoma of the colon: molecular pathogenesis and treatment. Anticancer Res. 2014;34(10):5517‐5521. 25275049

[20] Furlan D , Sahnane N , Mazzoni M , et al. Diagnostic utility of MS‐MLPA in DNA methylation profiling of adenocarcinomas and neuroendocrine carcinomas of the colon‐rectum. Virchows Arch. 2013;462(1):47‐56. 10.1007/s00428-012-1348-2. 23224118

[21] Tanaka T , Kaneko M , Nozawa H , et al. Diagnosis, assessment, and therapeutic strategy for colorectal mixed adenoneuroendocrine carcinoma. Neuroendocrinology. 2017;105(4):426‐434. 28641295 10.1159/000478743

